# Professionals’ perceptions of the establishment of a specialized brief therapy unit in a district psychiatric centre - a qualitative study

**DOI:** 10.1186/s12913-020-05926-8

**Published:** 2020-11-20

**Authors:** Hilde V. Markussen, Lene Aasdahl, Marit B. Rise

**Affiliations:** 1 Department of Mental Health, Faculty of Medicine and Health Sciences, Norwegian University of Science and Technology, Trondheim, Norway; 2 St. Olavs Hospital, Trondheim University Hospital, Nidaros District Psychiatric Centre, Trondheim, Norway; 3 Department of Public Health and Nursing, Faculty of Medicine and Health Sciences, Norwegian University of Science and Technology, Trondheim, Norway; 4 Unicare Helsefort Rehabilitation Centre, Rissa, Norway

**Keywords:** Mental health services, Short-term therapy, Health personnel, Organisational innovation, Mental health professionals, Implementation, Qualitative research

## Abstract

**Background:**

Increasing mental health problems and scarce treatment resources put pressure on mental health services to make innovations in service provision, such as developing differentiated services adapted to different needs. One innovation in differentiated service provision is brief or short-term treatment to patients with moderate mental health problems. Implementing a new unit in an organization usually faces many potential barriers and facilitators, and knowledge on how the professionals providing the services perceive the implementation of innovative approaches in mental health services is scarce. The aim of this study was therefore to explore the professionals’ perceptions of how the establishment of a specialized brief therapy unit had affected the organization, especially the everyday work in the outpatient clinics.

**Methods:**

Eleven professionals, five men and six women, took part in individual interviews. All participants were between 40 and 60 years old and had leading or coordinating positions in the organization. Their professional backgrounds were within psychology, nursing and medicine, most of them specialists in their field. Data was analyzed according to Systematic text condensation.

**Results:**

The professionals’ experiences represented four main themes: (1) The brief therapy unit was perceived as successful and celebrated. (2) The general outpatient clinics, on the other hand, were described as “forgotten”. (3) The establishment process had elucidated different views on treatment in the outpatient clinics - and had set off (4) a discussion regarding the criteria for prioritizing in mental health services.

**Conclusion:**

Providing targeted treatment to patients with moderate mental health problems, while having a concurrent aim to solve broader problems in mental health services, entails a discussion regarding resource use and the appropriate level of treatment provision. Professionals should be more involved when innovative efforts are implemented, and the criteria for success must be conceptualized and evaluated. Longitudinal research on the implementation of innovative efforts in the services should include professionals’ and service users’ perspectives.

## Background

Mental health problems and the demand on mental health services are increasing globally [1]. Yearly, almost 40% of the European population suffers from a psychiatric disorder [2, 3]. Implementing improved psychological treatments and innovative working processes in mental health services are needed to reduce the burden of mental illness [2–4], calling on the health sector to increase cost efficiency, quality of treatment and continual improvement [5, 6]. Scarce resources have led to a growing interest in evidence-based approaches in psychiatric and psychotherapeutic treatments that are cost-effective [7], contributing to a focus on innovation and flexibility. The development of innovative therapy approaches is expected to help reducing waiting lists, filling gaps in current services, standardizing information exchange and improving clinical pathways [8, 9]*.* Nevertheless, research has shown that there is little evidence that evidence-based treatments are adopted or successfully implemented in a context in an optimal way [10]. Research has pointed out several knowledge gaps in studies of implementing innovation in health care systems [11]. Improvement work in complex health organizations involves various potential challenges, such as heterogeneous patient groups, organizational differences and different cultures in the organization [12]. Ensuring that effective interventions are implemented has been identified as a priority task for mental health services [10]. Researchers have argued that the concept of “implementation results” depends on context and must be understood as different from the clinical treatment results following an intervention or a new treatment method [13].

In order to meet the increased demand for mental health services, a District Psychiatric Center (DPC) in Central Norway set an example of service innovation. This DPC was the setting for this study. From 2009 to 2014 the center had an increase of 42% in referrals of patients in need for outpatient treatment, and the number of patients who needed such treatment increased with over 43%. To respond to this challenge, the DPC in 2016 added a specialized outpatient unit to strengthen its general outpatient services. The objective of the establishment of the new unit was to provide brief and effective therapy for patients diagnosed with moderate affective and/or anxiety disorders, while also freeing resources in the general outpatient clinics for the patient groups with more extensive treatment needs [14].

Brief therapy is defined as an efficient mental health therapy approach, focusing on the present (“here and now”) and the patient’s strengths, and having a decisive approach [15]. Studies have shown that short-term psychotherapy is beneficial for adults with common mental disorders [16], and that short-term cognitive behavioral treatment (CBT) has a good effect on depression and anxiety [5, 17, 18]. Studies exploring the therapists’ perspectives show, on the other hand, skepticism regarding limitation of treatment, fearing that therapy becomes superficial and less client-centered [19, 20].

Although brief therapy is shown to be efficient for several patient groups [5, 16–18] this does not guarantee that knowledge will be successfully implemented in practice [10, 21]. Implementing a new service or unit in an organization usually faces many potential barriers and facilitators embedded in the characteristics of the new effort; the characteristics of the intervention, the external conditions, the internal environment, the characteristics of the individuals involved, and the implementation process [21, 22]. In health services, barriers to implementation seems to arise at several levels, such as at the patient level, at the treatment team level, at the organizational level and at the market or policy level [22]. In fact, one of the most critical issues in mental health services research is the gap between what is known about effective treatment and what is provided to consumers in routine care [10]. Context, complexity and process have been described as aspects that influence the outcomes from implementation efforts [13, 21]. Some studies infer implementation success by measuring clinical outcomes at the client or patient level, while other studies measure the actual targets of the implementation [13]. Researchers from different academic traditions generally conceptualize their topic for research of innovations in health service organizations in different ways [11]. In the present study, the conceptualizing of innovative efforts in a health organization was as follows: Innovation in health care service delivery and organization is defined as a novel set of behaviors, routines, and ways of working that are directed at improving health outcomes, administrative efficiency, cost effectiveness, or users’ experience and that are implemented by planned and coordinated actions [11]. Organizational culture is defined as sets of shared norms, values and beliefs that develop in an organization as the members interact with each other and their environment, and that are manifested through the members’ behavior and attitudes at work [23]. The establishment of a new treatment unit providing a specialized service, such as brief therapy, to a specific patient group, might thus influence the organization in several ways. Exploring the professional’s perceptions of what contributes to a successful implementation of new practices in a traditional organization is important [24], and studies of change and implementation show that implementation processes can be complicated and unpredictable and that they should be context-sensitive [24]. The aim of this study was therefore to explore the professionals’ perceptions of how the establishment of a specialized brief therapy unit had affected the organization, especially the everyday work in the outpatient clinics.

## Methods

This was a qualitative study including individual semi-structured interviews with professionals in a DPC. The study was conducted from October 2018 to February 2019.

### Study setting

In Norway, health care is organized at two levels, in the primary or secondary health services. The responsibility and supervision for most secondary services and the hospitals lies with Ministry of Health and Care Services, while the municipalities provide primary care services such as preventive services and general practitioners (GPs) [25]. Psychiatric care is organized as treatment wards in hospitals and as DPCs, the latter can be organized within a larger hospital. The Ministry of Health governs the DPC’s activities through the Regional Health Authorities in annual “letters of instruction” [26].

The DPC in the present study was part of a university hospital in Central Norway, producing 260 man-years (full-time equivalents) and being one of three similar DPCs in the hospital trust. The catchment area included approximately 110.000 persons in urban and semirural areas and parts of a large city. The DPC provided in-patient treatment, ambulatory services, and different types of outpatient treatment. Patients were mainly referred to the DPC from their GP, or by psychologists or psychiatrists in private practice. The DPC determined whether treatment was needed and offered a right to treatment within a specific time period (a waiting period guarantee). Before 2016, the DPC’s outpatient services was organized in three outpatient clinics, all with a generalist focus. In 2016, one of the general outpatient clinics was selected to provide brief (short-term) therapy in a delimited unit [14]. The unit encompassed 10.5 therapist positions specializing in short-term therapy, in contrast to the more generalist approach in the other general outpatient clinics in DPC. The unit’s target group was patients with anxiety and/or depressive disorders who previously had good functioning and self-esteem, but with a sudden fall in function, reactive states or sudden life events [14]. Treatment in the brief therapy unit was limited up to ten treatment sessions, individually or in groups, and included CBT, meta-cognitive therapy [27], mindfulness [28, 29] and Acceptance and commitment therapy [28].

### Participants and recruitment

Eligible participants in this study were leaders, coordinators and key personnel on different levels in the outpatient clinics at the DPC, as well as the head clinic leader and leaders of the mental health services at the hospital. The sampling aimed at recruiting participants that had experience with the implementation and operation of the brief therapy unit, and from cooperation tasks within the DPC’s outpatient services. Based on this sample strategy, initial study participants were suggested by the DPC’s management. The participants in this study were then asked directly by the first author (HVM) to participate in individual interviews. Subsequently, HVM recruited additional professionals to strengthen the diversity and representativeness of the sample. Here, we sought to include professionals with different experiences and roles, both inside the brief therapy unit and in the other parts of the outpatient services. Twelve participants were asked and consented to participate in the study. Due to illness, one of the informants was unable to participate, and the final study sample included 11 professionals. All participants received oral and written information about the study and signed a written consent before taking part in interviews.

### Data collection

Interviews were conducted using a semi-structured interview guide [30] where the researcher has some predefined questions or topics but then probes further as the participant responds. The interview guide was developed by HVM and MBR and contained a list of questions and topic areas that should be covered in the interviews. Representatives from the Competence Center for Lived Experience and Service Development in Central Norway provided useful input. The issues in the interview guide were prepared to explore the professionals’ experiences with innovation and new services in mental health care. When a district psychiatric center presented a new practice as an innovative effort, we found it interesting to gain insight into the practice’s impact on the organization and the extent to which the impact was consistent with the intentional change in the organization. The interview guide included topics and questions intended to allow the professionals to reflect on and discuss how the establishment of the brief therapy unit had influenced their work and the overall work in the DPC. Important themes were how the work at the DPC had developed during and after the establishment of the brief therapy unit, potential benefits and challenges herein, the overall quality of services, and the organizational culture and environment. The first author (HVM) conducted all interviews, either in the professional’s office or in a meeting room, according to the professional’s wish. The interviews lasted between 50 min to 1 h and 20 min. The interviewer (HVM) made notes during and after each interview. All interviews were audio recorded and transcribed verbatim by the first author (HVM).

As the aim was to explore the professionals’ experiences, we were cautious not to put up too strict boundaries for themes that came up in the interviews. While some topics identified in the first interviews were incorporated into the interview guide, the main issues remained constant. A new topic that was incorporated was the resource situation in the other outpatient units, and how the establishment had influenced the overall workload in the outpatient services.

### Data analysis

Data was analyzed according to Systematic text condensation (STC) [31]. The analysis was conducted in a group of three researchers with different backgrounds. HVM is a doctoral candidate in medicine with a professional background from medical sociology and community planning at MA-level*.* She has several years of experience from mental health hospital planning, including service innovation tasks within from this area of Norway. LA is a researcher and physician (specialist in physical medicine and rehabilitation). MBR is a professor in mental health work with a background from psychology and public health.

The data analysis was conducted according to the four steps in STC [31]. Firstly, the first author (HVM) listened to all audio recordings and read all transcripts several times to achieve an overall impression of the material. Each researcher then read the same three interviews and suggested preliminary codes for a coding list. The authors discussed codes and made a final coding list which was used in the next step. Examples of preliminary codes were “Better classification between patients needing short-term and long-term treatment”, “A good and delimited service for anxiety and depression”, “The general outpatient clinics were forgotten in this process”, and “Polarization in the professional environment – them and us”. Secondly, Mindjet MindManager (2017) was used by HVM as a tool to visualize the codes in each interview and for sorting meaning units. This was presented as a visualized mind map for each interview and shared with the other authors. After discussion of preliminary themes, all the interviews were coded by HVM and sorted in code-groups, such as “Prioritization of patients for two different services”, “New and celebrated treatment services in the brief therapy unit”, “Culture - working environment”, “Treatment length and coherent treatment” and “Quality of treatment”. Thirdly, the authors narrowed the code groups to fewer subgroups after several discussions. HVM formulated text condensations as texts amalgamating the meaning units of these subgroups [31]. In the fourth and final step themes were constructed, synthesized and validated through discussions in the author group. Empirical dimensions were formed for each interview and similarities across interviews were reflected in the themes and quotations.

The iterative analysis process continued until data reached a point of convergence, where four themes encompassed the most prominent of the material. The first author (HVM) also presented preliminary findings in meetings with two different research groups at NTNU. The researchers in these groups had complementary experiences and gave fruitful feedback and input during the analytic processes - input which was shared with the other authors. All three authors were continuously looking for alternative interpretations in several meetings and critical discussions, before agreeing on preliminary themes. The first author (HVM) summarized and decontextualized the text from the interviews that could illuminate the chosen codes and themes, focusing on the informant’s experiences. Finally, the authors discussed if the reduced text reflected the main topics in the data material. Quotes from the data material were chosen to elaborate and illustrate the results. They were translated by the third author (MBR) and checked and approved by the first (HVM) and second author (LA).

### Ethics

This study was approved by the Regional Committees for Medical and Health Research Ethics in Central Norway (2018/49/REK Midt). The participants in this study received oral and written information about the study and signed a consent form before taking part in interviews. The project was conducted in line with the Helsinki declaration (World Health Organization, 2010).

## Results

Eleven professionals took part in interviews, five men and six women, all between 40 and 60 years old and with more than 3 years of college/university education. All worked on different levels in the outpatient clinics at the DPC, five of them as leaders, while six had responsibility as coordinators. Their professional backgrounds were within psychology (8), nursing (2) and medicine (1), most of them specialists in their field.

According to the professionals, the establishment of the brief therapy unit was the answer to a growing crisis in the DPC, a crisis due to an increase in referrals but not in resources. They described that the DPC would not have tolerated this much longer, and something had to be done to relieve the pressure.

The results are presented as four themes: 1) Brief therapy provided by a celebrated unit, 2) The “forgotten” clinics, 3) Elucidating different views on treatment and 4) Influencing the criteria for prioritizing.

### Brief therapy provided by a celebrated unit

The brief therapy unit was described as a most welcome innovative effort, and many portrayed the brief therapy unit as successful and celebrated. Soon after the establishment, the brief therapy unit had also become an arena for trying out further innovative means, such as online-therapy. The professionals described that leaders and professionals from hospitals across the country came to visit and to learn from their creation of the unit. They elaborated that visitors were told about the beneficial changes in the DPC, such as the structured number of treatment hours, research-based and structured treatment methods, and shorter waiting times. The professionals described no doubt that the DPC treated far more patients after the implementation of the brief therapy unit, and that young patients with less severe diagnoses seemed to profit from this treatment approach. They also told about an internal evaluation report measuring clinical outcomes at the patient level, showing that patients could achieve good results with brief therapy. The results were so good that the management wanted to expand and develop the service further.

*I have noticed that the brief therapy unit is held up as a good example of success. Something excellent and good that we should be proud of.* (HP2)

Professionals within the brief therapy unit described a unique “team-feeling” among the staff, and a specialization of treatment approaches, compared to the general outpatient clinics. They elaborated that the unit had gathered a team of professionals who were interested in the same treatment approach and that the environment was inspiring, motivating, and energizing. The professionals who worked in the brief therapy unit described this as positive and beneficial, appreciating the possibility to specialize together with other professionals with the same interest. They also expressed feeling safe as fellow professionals in the team and said they helped and supported each other.

*It is an advantage to work in a similar manner and to have a shared professional profile. It gives us the opportunity to develop a specialist environment and be good at that specific service.* (HP4)

Several of the professionals working in other parts of the organization were more critical to the establishment of the brief therapy unit. They highlighted that the new unit was in a different building, geographically separated from the rest of the DPC, and that the unit had evolved into a separate unit without contact or collaboration with the other outpatient clinics. External research funding had also made possible more professional development in the new unit, compared to the general outpatient clinics. Several voiced a lack of integration between the new unit and the rest of the outpatient services.

### The “forgotten” clinics

While the brief therapy unit was described as an innovative and celebrated part of the DPC, the general outpatient clinics were described by several as “forgotten”. Professionals working here said that they had expected the implementation of the brief therapy unit to give them more room for working with the more complex patient cases. According to them, this had not happened. The work pressure had instead increased, and the establishment of the new unit had not led to the expected ease in workload. Several expressed that increasing referral volume had led to the brief therapy unit now treated the “easiest” cases, while the far more complicated and complex cases were allocated to the general outpatient clinics. The latter group demanded extra resources and time, and only a slow positive improvement could be expected. Many described that this led to fewer positive stories and experiences of success, leading to frustration among the professionals.

*We don’t see the success stories anymore. The stories that held us up … that we sometimes discharged a patient as recovered … we hardly see that anymore. Now we are overloaded [… ] we don’t have the success stories and we report it as a personal work environment problem. [The professionals] feel that they are not competent anymore.* (HP6)

Some of the professionals working in the general outpatient clinics said that the increased workload neither was anticipated nor acknowledged by the DPC’s management. In their view, the delimited treatment focus in the new unit had resulted in a more distinct focus on prioritization between demanding patients in the general outpatient services. In their view, “the rest of the organization” had not been properly involved in the innovative approaches and development of better services. They missed that the management focused on the work and increased effort in the general outpatient clinics. The professionals pointed to the wide range of tasks in the general outpatient clinics and said that it was nearly impossible to keep updated, professionally and methodically, to handle the different and complex diagnoses. Several said that they missed consideration and recognition of the various disciplinary approaches, and that they had too little time to meet the needs of different patient groups. Some described a fear of having to schedule more infrequent treatment sessions for patients and to terminate treatment too early.

*I think the reason is that we cannot influence how many patients we receive […] and to manage [the case load] we “dilute” [the treatment]. This is against professional advice … and I think that professionals from different traditions experience this as a problem. Individual professional has too many patients on the list … more than they can manage. (HP7)*

### Elucidating different views on treatment

The establishment of the brief therapy unit also seemed to have highlighted the existence of different views on treatment within the DPC, namely on what constitutes good treatment. While some professionals highlighted short-term treatment as a success and a promising approach for the future, others voiced concerns about how focusing on short-term therapy could result in poorer treatment for patients with more complex needs. The professionals who were most positive to the short-term approach emphasized that the brief therapy unit was a positive addition to the outpatient treatment, providing targeted treatment to a large and increasing patient group. They attributed this to the DPC’s young patient population and said that targeting the youngest adults could have significant long-term benefits for the DPC. According to them, the implementation had provided a possibility for young adults to come early in contact with the mental health services, receiving targeted treatment quickly and, potentially, returning rapidly to society.

The more critical professionals said that young adults with mental health problems potentially received too limited treatment in their first meeting with psychiatry. They were concerned that all new patients struggling with anxiety and depression now received the same treatment approach, and that short-term treatment had become “the quick and only option” for a large group of young adults. Several stated that the establishment of brief therapy in the DPC was an expression of a trend towards attempting to resolve mental problems or disorders as quickly as possible.

*[Brief therapy] can be at the expense of thoroughness … making you lose eye with the underlying … and if you are focused on quick improvement, it governs the way we view a person, view the patient, understand the patient … In my opinion, it could be a risk.* (HP7)

According to some, the brief therapy unit had cultivated a standardized working method in “a one-sided manner”, describing this as an expression of a “quick fix”. Others said that while the management tried to handle the increased volume of referral, they forgot the patients with complex needs. In their view, the short-term approach was not sufficient to provide good treatment to the general patient population, since many patients would not benefit from standardized or time limited treatment.

*[The development of time limited treatment] has an unintended effect. The development I am talking about here is that we are expected to provide good services to more people with fewer resources. It is not possible to give good therapy to all in so short time. When this is presented as the solution to a much bigger problem, I become doubtful. We use internal resources to focus more on time limited treatment, resources that could have been used for patients with more complex needs. *(HP6)

### Influencing the criteria for prioritizing?

Many of the professionals discussed whether the development towards more short-term approaches influenced the criteria for prioritizing in the mental health services. The focus on young adults with anxiety and depression, was described as a potential driver for lowering the threshold for treatment in the DPC. Some said that the threshold had already been lowered after the implementation of brief therapy, resulting in more referrals of patients with less severe diagnoses. Others claimed that the patient population in the DPC had changed over two decades, and that an increasing group of younger patients with moderate problems demanded a larger share of the resources. Some said that society was responsible for handling and normalizing some of the mild mental challenges the patients experienced, and that referring and providing treatment to all types of mental problems was neither sustainable nor appropriate.

*We tend to “therapeuticize” needs in people. I think this is part of the explanation for the large group we shall manage. That we over-use therapy.* (HP5)

The professionals also attributed a potential lowering of the threshold for treatment in the specialized services to the current priority guidelines in the mental health services. Some said that they had to balance what they perceived as conflicting guidelines: To prioritize between patients and at the same time reject fewer, describing this as an impossible task. Many emphasized that the government’s guidelines, stating that youth should be prioritized, probably resulted in more young people with moderate diagnoses being offered treatment in the DPC.

*One the one hand we are supposed to prioritize. On the other hand, we have a minister of health that gets a tummy ache thinking of someone who will be rejected. So, we should meet everybody and be available, but we also must prioritize. It does not add up.* (HP1)

Professionals discussed the future of the DPC and how the system could handle the increasing number of patients in need of treatment. According to some, treating more patients with less severe diagnoses implied doing the work for primary care, thus affecting the treatment of patients with more complex problems who should be the most important group for the DPC. Several were concerned that the resources were used incorrectly, and that moderate mental problems should have been treated elsewhere. In their view, the general increase in mental health problems, particularly among young people, should have resulted in more responsibility for these patients within other parts of the health care system, such as the student health services and the municipal health services. Some said that a general patient admission across service levels could improve this situation. They advocated the establishment of an interdisciplinary team for improving the prioritization of patients between different levels of mental health services.

*The more we establish frontline services like this, the more we undermine the expectations that the municipality ... primary care, should be managing these patient groups.* (HP6)

Some stated that it was a misconception that the brief therapy unit treated only moderate problems. In their opinion, the patients were too sick to receive treatment at the primary care level and that the brief therapy approach mainly had contributed to more differentiation of the services and thus more targeted treatment in DPC.

*The cases are not mild, that is a myth. When we look at the diagnoses they have [ …] not only anxiety and depressions. They have other types of problems as well. They have recurrent depressions; they have personality problems. I don’t know whether they are very different from the patient population in the general outpatient clinics, except for the comprehensive and complex cases where it is obvious that ten sessions are not sufficient. [ …] Besides that, I do not think the patient population is very different.* (HP9)

## Discussion

### The shift towards short-term treatment

In the present study, the establishment of a brief therapy unit was described as complying with treatment guidelines provided by the health authorities, encouraging more innovation to provide treatment to a larger number of patients without any increase in resources. The establishment of the new unit was therefore perceived as an important signal that the DPC recognized and followed up the expectations from health authorities. Authors have argued that third parties, such as health authorities and insurers have an increasing influence on mental health services, for example in limiting the amount of treatment [7, 20]. In the present study, the health authorities were understood as such a third party, strongly influencing the treatment provided in the outpatient services. This finding contributes to an understanding of how policy guidelines can affect both problem definition and approach [20] during the implementation of innovation tasks and the development of treatment alternatives in mental health services. The trend of short-term treatment was also viewed differently within the DPC. For some, shorter treatment represented a positive influence by drawing attention to effective and adequate treatment. Others argued that brief therapy represented a somewhat superficial approach to complex patient needs. Both previous and current mental health care encompass different schools of thought with different perspectives on how to explain and treat mental illness [7]. Different views in the present study might thus be an expression of a complex organizational culture [23] encompassing therapists with different professional training and backgrounds. Implementation of innovative treatment offers is by nature a social process that is intertwined with the context in which it takes place [22] and our findings according to the internal environment in the DPC might shed light on a possible organizational barrier to implementation.

The described trend of short-term treatment also seemed to challenge the autonomy, independence and judgement of some of the professionals, as well as inducing a risk of burnout for the professionals working in the general outpatient services. This is in line with previous research, suggesting that the regulation that lies within bureaucratization and national governance weakens medical professionals’ autonomy [32]. Standardization of services, such as time limitations, can lead to professionals’ skepticism and resistance [7, 12]. Burnout in doctors and other health professionals results in negative effects on patient care, professionalism, therapists’ self-care, and the health care system’s viability [33] Research indicates that both individual-focused and structural or organizational strategies can result in reductions in the experience of burnout [34] and such strategies can facilitate a more successful implementation of service innovation. Further research should examine the complex ways in which organizational culture and different strategies can influence professionals’ attitudes to innovation efforts in health organizations and how this can possibly be better accommodated in practice.

Several have described prerequisites for successful implementation of new efforts [22, 34]. Different perceptions of the benefits of the innovation, the consensus among professionals and the interactions between innovation and the context influences the innovative process [35]. One aspect is the importance of collective action; that all participants agree on the implementation of the new effort and are willing to contribute in the work [36]. Including all stakeholders in planning and preparing is thus recommended before implementing changes [36]. In addition, management has an important role in innovation processes, especially in maintaining a positive relationship with employees [35]. To provide interaction between professionals at different levels in the organization, management should engage in extensive information sharing across organizational levels by applying a bottom-up approach, two-way communication and feedback, including evaluation, modification and improvement of innovative efforts [35]. Investing more in securing the staff’s commitment before and during implementation of new efforts would thus be useful. This includes asking for input from the staff and using this information to adjust the course during the implementation process. Another important role in implementation processes is the middle manager. In the present study, some of the leaders and coordinators at different levels expressed that they had not been properly involved in the innovation process. Middle managers might influence the implementation of healthcare innovation by disseminating information, synthesizing information, communicating between strategy and daily activities among employees [37]. Involving the professionals on different levels in larger part of the organization in the implementation process might thus have been helpful. Further studies should investigate the middle managers role in improving the implementation of mental healthcare innovation.

### Success depends on what you are looking for

In the present study, the brief therapy unit was established to target a growing patient group, namely young adult patients with anxiety and/or depression. However, an additional aim was to free up resources in the general outpatient clinics for the patient groups with more extensive treatment needs. The results reflected this twofold aim, showing two different views on whether the innovative effort was a success or not. While most agreed that the brief therapy unit provided good treatment outcomes for the targeted patient group, an outcome that encouraged the management to expand and develop further the brief therapy service, the more critical voices put emphasis on other influences from the implementation, such as more pressure on the general outpatient units and not enough focus on patients with more extensive treatment needs. An important issue in implementation of innovative efforts is how to conceptualize and evaluate success [13]. The concept of “implementation results” depends on context and must be understood as different from the clinical treatment results following an intervention or a new treatment method [13]. A distinction should thus be made between considering a new measure as a limited intervention or as an organizational innovative effort with more goals. It is therefore reasonable to ask whether patients with complex mental health problems and associated services in the DPC received sufficient attention in the innovation work that took place. In this context, it is important that policymakers, service providers, and mental health professionals to understand the very different needs in the patient population when facilitating innovational change in specialist health care such as DPC.

Studies of implementation have used varying approaches [13]; some measuring clinical outcomes at the patient level, while others have measured the organizational or broader targets of the implementation [10]. However, implementation of any new treatment or service is recognized as a process that involves a variety of activities in an organization [13]. In the DPC investigated in the present study, it might have been useful to visualize the establishment of the new unit as part of a broader strategy demonstrating the organization’s effort to improve broader aspects of the mental health services.

### Providing mental health care on the appropriate level

On what care level common mental health problems should be treated is an ongoing discussion regarding accurate identification of problem, appropriate treatment, costs, and waiting time [38]. Providing mental health treatment in primary care should secure treatment that is effective, efficient, accessible, and equitable [38]. The present study showed that several of the professionals meant that young adults with moderate mental health problems such as anxiety and depression, should not be treated in the DPC, but rather be the responsibility of primary care services. This is in line with one of the main aims of the Norwegian Coordination Reform [39] requiring a transferal of tasks and responsibility to the primary health services. Similarly, The World Health Organization has stated that mental health treatment should take place in primary care [40].

Whether mental health care is provided mainly at the primary or secondary care level varies a lot in Europe [41]. Researchers have claimed that collaboration between the primary and secondary mental health services is needed to provide care across systems [42], and that a shift in resource balance between the care levels is needed [43]. Collaboration between primary and secondary health services is also emphasized and advised in treatment guidelines for common mental health treatment [38, 44], and several models have been suggested for closer integration and collaboration [45]. Such models include training of primary care professionals, consultation-liaison collaboration (where a secondary care specialist provides support during care whenever needed), collaborative care (where appointed care managers secure a collaboration between primary and secondary care professionals), and referral (where the patient is referred to secondary care for treatment) [45]. Collaborative models for treatment of depression and anxiety disorders in primary health care have shown to be effective and providing mental health care at this level is cost-effective [42, 46, 47].

Similarly, treatment in primary care with a permanent care manager can be positive and represent continuity for patients who need long-term treatment and follow-up [48]. In addition, brief psychological treatment approaches (CBT, counselling and problem-solving therapy) can be provided effectively in primary health care [49]. Although the present study showed disagreement between the professionals about the severity of the patients’ illness, patients with moderate anxiety and/or depression could thus be successfully treated in or in some type of collaboration with primary care. Future studies should thus investigate on what treatment level young adults with moderate anxiety and/or depression could be provided short-term treatment in the most cost-effective way and with the best treatment outcome. Studies comparing similar treatment in primary or secondary care level, respectively, could answer this question.

### Strengths and limitations

The study sample consisted of leaders, coordinators and other key personnel. The sample had good gender representation and diversity of experiences from different parts of the outpatient clinics in the DPC. This provided data material that reflected the width of professionals’ experiences and strengthens this study. The first participants were identified and suggested by the DPC’s management. Subsequently, HVM recruited more participants to include various perspectives. Asking leaders to recruit study participants brings about potential bias, since they influence the choice of information sources. Nevertheless, the study depended on interviewing professionals who had first-hand experience with the establishment of the new unit, and who could view this from different positions in the organization. We therefore found the recruitment process adequate.

A qualitative semi-structured interview approach made it possible to explore the professionals’ individual experiences without setting boundaries for the themes brought up during interviews. This strengthened the exploratory approach. While an exploratory approach allows for all types of experiences and perceptions, this also imply limitations. Most of the results convey the professionals’ personal experiences and points of view and are not confirmed by any other types of data. Many of the findings must therefore be interpreted with caution. One example is the statement that the general outpatient clinics were forgotten and not acknowledged by the DPC’s management. We have no other data material confirming these statements. Conducting interviews at a single time point also includes the risk of recall bias. Data collected on different time points in the implementation process would have strengthened the long-term perspective. Encompassing the longitudinal approach should be pursued in further research on innovative efforts in mental health settings.

The analysis was conducted by three authors with backgrounds in social science/public health, medicine and psychology. The first author (HVM) is educated as community planner with several years of experiential knowledge from mental health hospital planning including service innovation tasks. The 3rd author (MBR) has extensive experience with qualitative analyses, and the 2nd author (LA) has some experience. The diversity in backgrounds and experience is a strength in this study. The author group had several meetings, continuously looking for alterative interpretations before agreeing on every step in the analysis process. To allow for alternative understandings and perspectives on the data material, preliminary results were discussed several times in two separate research groups, one group with an exclusively qualitative methods approach, and one with a more comprehensive methodological focus. This helped provide alternative points of view in the analysis process and strengthens the study.

The Norwegian socio-cultural context, such as the organization of mental health care and the comprehensiveness of the welfare system, somewhat limits the transferability to other countries. The present study did not measure treatment outcome or patients’ experiences. Neither can we be certain whether the professionals’ perception of increasing time constraints was a result of the establishment of brief therapy or part of a general development in the mental health services. Other types of studies are needed to investigate this.

## Conclusion

This study explored professionals’ experiences with the establishment of a new unit providing short-term therapy in the outpatient services of a DPC. The results showed that, while being in line with health authorities’ treatment guidelines, the trend of short-term treatment challenged the professionals’ autonomy and judgment. Offering targeted treatment to patients with moderate mental health problems, while having a concurrent aim to solve broader problems in mental health services, entails a discussion regarding resource use and the appropriate level of treatment provision. As contemporary mental health policy focuses on promotion, prevention and early intervention as innovative efforts, our study shows that professionals call for the authorities’ focus on the ongoing needs of those with longer-term and more complex mental health problems. Choice of focus during innovation and implementation processes might influence what is given attention. There might thus be a need for a more complete system approach to prevention, treatment and rehabilitation for the heterogeneous patient population who need different mental health services.

To improve implementation processes in mental health care, professionals from different levels of the organization, as well as the primary care level, could be more involved. The aims of innovative efforts are not always clear-cut, and success needs to be conceptualized and evaluated. Further research should include a longitudinal perspective on the implementation of innovative efforts in the services, including the perspectives from leaders, professionals and service users in various parts of the services.

## Data Availability

The datasets generated and analyzed during the current study are not publicly available due to ethical approval but are available from the corresponding author on reasonable request.

## Abbreviations

DPC: District Psychiatric Center
GP: General Practitioner
NTNU: Norwegian University of Science and Technology
